# Atypical fractures at non-classical sites associated with anti-resorptive therapy: a systematic review

**DOI:** 10.1093/jbmr/zjae159

**Published:** 2024-09-30

**Authors:** Lucy Collins, Alec Ronan, Evelyn Hutcheon, Peter R Ebeling, Vivian Grill, Hanh H Nguyen

**Affiliations:** Department of Medicine, School of Clinical Sciences, Monash University, 246 Clayton Road, Clayton, VIC, 3168, Australia; Department of Endocrinology and Diabetes, Western Health, 176 Furlong Road, St Albans, VIC, 3021, Australia; Department of Endocrinology, Monash Health, 246 Clayton Road, Clayton, VIC, 3168, Australia; Department of Endocrinology and Diabetes, Western Health, 176 Furlong Road, St Albans, VIC, 3021, Australia; Western Health Library Service, 176 Furlong Road, St Albans, VIC, 3021, Australia; Department of Medicine, School of Clinical Sciences, Monash University, 246 Clayton Road, Clayton, VIC, 3168, Australia; Department of Medicine, The University of Melbourne, 161 Barry Street, Carlton, VIC, 3010, Australia; Department of Endocrinology and Diabetes, Western Health, 176 Furlong Road, St Albans, VIC, 3021, Australia; Department of Medicine, The University of Melbourne, 161 Barry Street, Carlton, VIC, 3010, Australia; Department of Medicine, School of Clinical Sciences, Monash University, 246 Clayton Road, Clayton, VIC, 3168, Australia; Department of Endocrinology and Diabetes, Western Health, 176 Furlong Road, St Albans, VIC, 3021, Australia; Department of Endocrinology, Monash Health, 246 Clayton Road, Clayton, VIC, 3168, Australia; Department of Medicine, The University of Melbourne, 161 Barry Street, Carlton, VIC, 3010, Australia

**Keywords:** atypical fracture, anti-resorptive therapy, bisphosphonates, denosumab

## Abstract

Osteoporosis is common, affecting more than 20% of women and 6% of men globally over the age of 50. Anti-resorptive drugs, bisphosphonates and denosumab, have been effective treatments for osteoporosis for more than 30 years. Rare complications of anti-resorptive therapy include medication-related osteonecrosis of the jaw and atypical femur fractures (AFF). The American Society for Bone and Mineral Research (ASBMR) proposed a case definition for these atypical femoral fractures in 2010, which was updated in 2013. However, atypical fractures at non-classical sites have been increasingly described. We aimed to systematically identify atypical fracture cases excluded from the ASBMR AFF case definition in patients receiving anti-resorptive medication for longer than 3 yr. A structured search of electronic databases, including PubMed, Medline (Ovid), Embase (Ovid), Cochrane, and Web of Sciences, and hand-searching of conference abstracts were undertaken. All full-text articles written in English describing an atypical fracture in patients (aged >18 yr) and receiving anti-resorptive medication for >3 yr were included, with data extracted and analyzed by two independent reviewers. Sixty-six articles were identified, describing 151 cases of atypical fractures in 114 individuals. The most frequent fracture site was the ulna, followed by the tibia. All patients were taking anti-resorptive treatment prior to or at the time of fracture, most frequently alendronate monotherapy (44%). Most commonly, fractures were transverse in nature (95%), following minimal or no trauma (96%), and non-comminuted (98%) with cortical thickening of the surrounding bone (69%). Anti-resorptive treatment was ceased following an atypical fracture in the majority (89%). Atypical fractures are rare and should not deter physicians from appropriate anti-resorptive therapy for osteoporosis. However, clinicians should be alert to their presence at additional sites to the femur. An update of the current ASBMR AFF case definition to include other skeletal sites could be timely.

## Lay Summary

Bisphosphonates and denosumab, anti-resorptive medications, have been effective treatments for osteoporosis for more than 30 yr. Rare but serious side effects of these therapies include medication-related osteonecrosis of the jaw and atypical femur (thigh bone) fractures. Increasingly, however, atypical fractures at non-classical sites have been described. To address this, we systemically reviewed published cases of atypical fractures at sites other than the femur. We identified 151 cases of atypical fractures in 114 individuals. The most frequent site was the ulna, followed by the tibia. All patients were taking anti-resorptive medication prior to or at the time of fracture, most frequently alendronate (44%). Most commonly, fractures were transverse in nature (95%), following minimal or no trauma (96%), and non-comminuted (98%) with cortical thickening of the surrounding bone (69%). Although rare, clinicians should be alert to the presence of atypical fractures at sites additional to the femur. If confirmed to be an atypical fracture in the setting of anti-resorptive therapy, we recommend considering cessation of anti-resorptive treatment, limiting weight-bearing, considering surgical management, and managing their underlying osteoporosis.

## Introduction

Osteoporosis is common, affecting more than 20% of women and 6% of men globally over the age of 50.^1^ Osteoporotic fractures are highly significant, leading to persistent pain and increased mortality of over 30% within 1 yr following hip fractures.^2^ Fragility fractures are estimated to cost the healthcare system more than €56 billion per annum in Europe alone.^3^ Oral and intravenous bisphosphonates are anti-resorptive drugs that have been the most used treatments for osteoporosis globally for more than 30 yr. Bisphosphonates reduce the risk of vertebral fractures in older men and vertebral and nonvertebral fractures in postmenopausal women with osteoporosis.^4–6^ Another widely used anti-resorptive drug is denosumab, a human monoclonal antibody specifically targeting the RANK ligand. Denosumab inhibits osteoclast function, maturation, and survival, thereby reducing bone resorption and has been shown to reduce vertebral, non-vertebral, and hip fractures in women with postmenopausal osteoporosis.^7^

Despite the availability of effective anti-resorptive therapy, treatment rates have declined following reports of rare side effects, specifically medication-related osteonecrosis of the jaw and atypical femur fractures (AFFs).^8^ AFFs were first reported in 2005 by Odvina and colleagues, highlighting a state of significantly suppressed bone turnover on bone biopsy.^9^ The case series described nine patients on long-term alendronate treatment (3-8 yr) who sustained spontaneous non-spinal fractures while on anti-resorptive treatment, including five femur fractures. However, insufficiency fractures were identified at other sites, including the pelvis, sacrum, vertebra, rib, and metatarsal. In 2012, a systematic review and meta-analysis found that bisphosphonate exposure was associated with an increased risk of subtrochanteric and femoral shaft fractures with an adjusted relative risk of 1.70 (95% confidence interval, 1.22-2.37).^10^ In 2010, the ASBMR Task Force proposed a case definition for these atypical fractures affecting the femoral diaphysis.^11^ The definition was updated in 2013^12^ (Table 1).

**Table 1:** ASBMR Task Force Revised Case Definition of AFFs.^12^

Definition: To satisfy the case definition of AFF, the fracture must be located along the femoral diaphysis from just distal to the lesser trochanter to just proximal to the supracondylar flare. In addition, at least four of five Major Features must be present. None of the Minor Features is required but have sometimes been associated with these fractures.	
**Major features**	The fracture is associated with minimal or no trauma, as in a fall from a standing height or less
	The fracture line originates at the lateral cortex and is substantially transverse in its orientation, although it may become oblique as it progresses medially across the femur
	Complete fractures extend through both cortices and may be associated with a medial spike; incomplete fractures involve only the lateral cortex
	The fracture is noncomminuted or minimally comminuted
	Localized periosteal or endosteal thickening of the lateral cortex is present at the fracture site (“beaking” or “flaring”)
**Minor features**	Generalized increase in cortical thickness of the femoral diaphysis
	Unilateral or bilateral prodromal symptoms such as dull or aching pain in the groin or thigh
	Bilateral incomplete or complete femoral diaphysis fractures
	Delayed fracture healing

Certainly, in the literature, the majority of atypical fractures affect the femur. However, case reports of fractures at other skeletal sites with atypical features like AFFs have been reported but are excluded from the ASBMR case definition. Such fractures have been periprosthetic, located at non-femoral sites, or medially located along the femoral diaphysis.^13^^,^^14^ Although rare, atypical fractures not fulfilling the ASBMR criteria may provide insight into AFF pathogenesis. In 2015, Tan and colleagues published the first systematic review of atypical ulnar fractures.^14^ Recently, in 2021, De Cicco and colleagues systematically reviewed case reports of atypical periprosthetic fractures.^13^ To date, however, no systematic review has addressed atypical fractures located at non-classical sites in totality. This study aimed to systemically identify and characterize atypical fractures cases, excluded from the current ASBMR AFF case definition, in patients receiving anti-resorptive therapy for longer than 3 yr.

## Materials and methods

**Figure 1:**
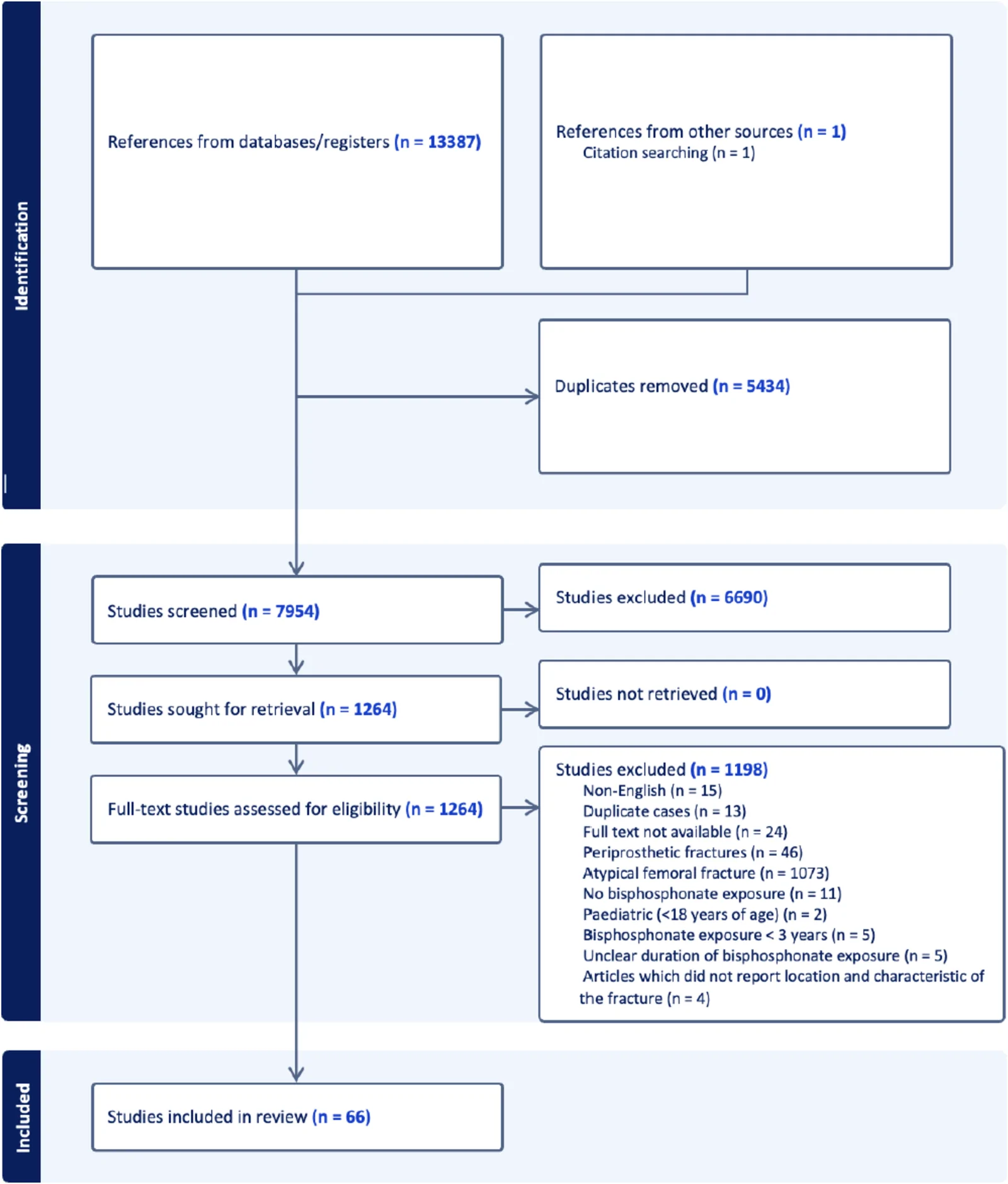
Flow diagram detailing article selection.

This review was conducted according to the PRISMA guidelines (PROSPERO CRD42023424677). A structured search of electronic databases was conducted, including PubMed, Medline, Embase, Cochrane, and Web of Science on 21 October 2022, and hand-searching of conference abstracts/reference lists thereafter (Supplementary 1). The following key words were used: “bone density conservation agents” OR “antiresorptive” OR “diphosphonates” OR “denosumab^*^” OR “alendronate^*^” OR “ibandronic acid^*^” OR “pamidronate^*^” OR “zoledronic acid^*^” OR “risedronic acid” OR “bisphosphonate^*^” OR “prolia” OR “xgeva” OR “zoledronate^*^” OR “zometa” OR “aclasta” OR “risedronic acid^*^” OR “actonel” OR “risedronate^*^” OR “atelvia” OR “ibanaronate^*^” OR “bondronat” OR “boniva” OR “bonviva” OR “romosozumab^*^” AND “fractures” AND “atypical” OR “insufficiency” OR “stress”. The articles were reviewed in two stages. First, the abstracts of all citations identified were screened by two reviewers (LC and AR) based on the following inclusion criteria: (1) cases of atypical fractures in patients (aged >18 yr) receiving long-term anti-resorptive therapy (>3 yr) and (2) cases published 2005–2022. Atypical fractures were defined as insufficiency fractures following minimal or no trauma, which may be incomplete or complete, transverse in nature with minimal comminution, and associated with cortical beaking. Any points of difference were reviewed by a third independent reviewer (H.H.N.). Following, all full-text articles identified using the inclusion criteria written in English language describing atypical fractures were screened for eligibility by two independent reviewers, L.C. and A.R. Exclusion criteria included non-English, duplicate cases, full text not available, unclear duration or <3 yr of anti-resorptive therapy, pediatric population, articles that did not report location/characteristics of the fracture, and AFFs fulfilling the second ASBMR Task Force case definition.^12^ Given the recent systematic review by De Cicco and colleagues, periprosthetic AFFs were excluded from the search.^13^ Figure 1 illustrates the selection process. Two authors, L.C. and A.R., extracted the data independently from the included articles. Demographic variables (age, sex), medical co-morbidities, ambulatory status, anti-resorptive therapy details (dose, frequency, duration, cessation with fracture), fracture characteristics, and fracture management were recorded. Results from laboratory investigations and DXA were collected if available. Any points of difference between L.C. and A.R. were resolved following review and discussion. The quality of the included articles (predominantly case reports) was assessed using one of the few tools available—the Joanna Briggs Institute (JBI) Critical Appraisal Checklist for Case Reports.^15^

Fracture characteristics were assessed via two methods. First, reported data were extracted from the articles included in this review. Fracture characteristics included partial/complete fracture, absence/presence of comminution, transverse fracture, characteristics of surrounding bone (ie, cortical thickening, sclerosis, and bone oedema), and presence/absence of a “beak sign” involving the lateral cortex. Second, authors L.C. and A.R., independently reviewed radiographs (if published) and recorded the following fracture characteristics: partial/complete fracture, transverse fracture, absence/presence of cortical spike, and origin of the fracture site. Fracture site, association with trauma, prodromal symptoms, fracture pattern, treatment, and delayed healing were also extracted.

Descriptive data are presented as means, median, and range unless stated otherwise.

## Results

Using the search strategy outlined above, 7954 citations were identified, and 1264 articles fulfilled the inclusion criteria. Upon full-text review, 1198 articles were excluded. During the preparation of this review, a further relevant article was published and also included. Therefore, a total of 66 articles were analyzed (see Supplementary Material),^9^^,^^14^^,^^16–48^ including 54 case reports, 7 case series, 3 case control trials, 1 cohort study,^17^ and one systematic review.^14^ Articles describing the same patient cohort were only counted a single time.^14^^,^^25^

A total of 151 cases of atypical fractures were reported in 114 individuals. Most atypical fractures occurred in females (*n* = 99, 91%), with a median age of 71 yr (IQR 63-78) (Table 2). The most frequent fracture site was the ulna (*n* = 59 fractures; 53 individuals), followed by tibia (*n* = 15 fractures; 12 individuals), metatarsal (*n* = 15 fractures; 12 individuals), vertebrae (pedicle) (*n* = 12 fractures; 6 individuals), pelvis (*n* = 12 fractures; 12 individuals), sacrum (*n* = 10 fractures; 8 individuals), femoral neck (*n* = 6 fractures; 5 individuals), radius (*n* = 5 fractures; 5 individuals), humerus (*n* = 4 fractures; 4 individuals), fibula (*n* = 3 fractures; 2 individuals), scapula (*n* = 2 fractures; 2 individuals), distal medial femoral shaft (*n* = 2 fractures; 2 individuals), rib (*n* = 2 fractures; 2 individuals), clavicle (*n* = 1 fracture; 1 individual), femoral head (*n* = 1 fracture; 1 individual), vertebra (site not specified) (*n* = 1 fracture; 1 individual), and sternum (*n* = 1 fracture; 1 individual).

**Table 2:** Patient characteristics.

**Sex (*n* (%))**	
** Female**	99 (91%)
** Male**	10 (9%)
**Age (y, median (IQR))**	71 (63-78)
**Fracture site (*n* (%))**	
** Ulna**	59 (39.1%)
** Tibia**	15 (9.9%)
** Metatarsal**	15 (9.9%)
** Vertebrae (pedicle)**	12 (7.9%)
** Pelvis**	12 (7.9%)
** Sacrum**	10 (6.6%)
** Femoral neck**	6 (4.0%)
** Radius**	5 (3.3%)
** Humerus**	4 (2.6%)
** Fibula**	3 (2.0%)
** Scapula**	2 (1.3%)
** Distal medial femoral shaft**	2 (1.3%)
** Rib**	2 (1.3%)
** Clavicle**	1 (0.6%)
** Femoral head**	1 (0.6%)
** Sternum**	1 (0.6%)
** Vertebrae (location not specified)**	1 (0.6%)
**Bilateral fractures reported (*n* (%))**	28 (19%)
**Type of anti-resorptive therapy (*n* (%))**	
** Alendronate**	50 (43.9%)
** Zoledronic acid**	6 (5.3%)
** Risedronate**	5 (4.4%)
** Ibandronate**	5 (4.4%)
** Denosumab**	1 (0.9%)
** Pamidronate**	1 (0.9%)
** Etidronate**	1 (0.9%)
** Sequential**	19 (16.7%)
** Specific type not reported**	26 (22.8%)
**Indication for anti-resorptive therapy (*n* (%))**	
** Osteoporosis**	96 (84.2%)
** Malignancy**	9 (7.9%)
** Not reported**	9 (7.9%)
**Duration of anti-resorptive treatment (y, median (IQR))**	8 (5.6-10)
**Associated monogenetic bone disorder (*n* (%))**	
** Yes**	1 (0.9%)
** Hypophosphatasia**	1 (0.9%)
** No**	113 (99.1%)
**Oral/intravenous corticosteroid use >3 mo duration (*n* (%))**	
** Yes**	8 (7.0%)
** No**	106 (92.9%)

Of the 114 individuals, 96 (84%) patients had a history of osteoporosis, and 9 individuals (8%) commenced anti-resorptive therapy in the setting of malignancy (metastatic breast carcinoma,^39^ metastatic follicular thyroid carcinoma,^37^ giant cell tumor of bone,^33^ and multiple myeloma^46^). None of the atypical fractures in these cases were pathological fractures due to metastatic disease. Murai and colleagues completed an ^17^FDG-PET/CT to exclude a pathological fracture.^37^ There was one report of an atypical tibial fracture in a female with an underlying monogenetic bone disorder (hypophosphatasia) who had received 4.5 yr of intermittent alendronate therapy.^34^

All patients were taking anti-resorptive therapy prior to/at the time of the atypical fracture, with a median duration of anti-resorptive therapy of 8 yr (IQR 5.6-10) (Table 2). Out of 66 articles, 45 reported data on cessation of anti-resorptive therapy following atypical fracture. The majority ceased treatment (*n* = 40, 89%); however, five cases did not.^22^^,^^37^^,^^39^^,^^43^^,^^44^ The specific anti-resorptive agent used was specified in 77% of cases (88 individuals). Alendronate monotherapy was the most frequent agent (*n* = 50, 44%). Only one case of denosumab monotherapy was reported: a case of bilateral tibial stress reactions while receiving denosumab 120 mg monthly for 4 yr for the treatment of a giant cell tumor of bone.^33^ Five cases elected to pursue denosumab treatment following an atypical fracture. In three cases, bisphosphonate treatment was changed to denosumab following an atypical fracture.^39^^,^^43^^,^^44^ On the other hand, Binkley et al., and Murai et al., elected to continue denosumab treatment following atypical fracture.^22^^,^^37^

Most fractures were sustained following “minimal or no trauma, such as a fall from a standing height or less” (*n* = 110, 96%). One case occurred following a fall that did not specify the degree of trauma,^16^ and another occurred following almost tripping and hitting their leg against a stair banister.^23^ The presence or absence of prodromal symptoms were commented on in 102 fractures; 63 (62%) fractures were preceded by prodromal pain, while 39 (38%) fractures were silent (Table 3, see Supplementary Material). Of the total 59 ulnar fractures included in this review, only five articles (describing six fractures) commented on ethnicity; five ulnar fractures occurred in individuals of Asian ethnicity (Thai, Chinese, and Japanese), and one ulnar fracture occurred in a White individual.

**Table 3:** Atypical fracture characteristics.

				**Fracture characteristics as described in article**				
**Author (Year)**	**Site of fracture**	**Prodromal pain**	**X-ray included in report**	**Incomplete/complete**	**Fracture comminution**	**Characteristics of surrounding bone**	**“Beak sign” of lateral cortex**	Fracture radiograph pattern^*^
**Kim (2012) (see Supplementary Material)**	Right femoral neck	Yes	Yes	Incomplete	No	Sclerosis	No	Partial, transverse
**Tang (2011)^45^**	Right proximal ulna Right proximal tibia	Yes Yes	Yes Yes	NR NR	No No	NR NR	NR NR	Complete, transverse Partial, transverse
**Gharanizadeh (2023) (see Supplementary Material)**	Type 1 sacrum Acetabulum with femur head protrusion into the pelvis Quadrilateral surface fracture Right anterior column Right superior and inferior pubic fracture	Yes Yes Yes Yes Yes	Yes Yes Yes Yes Yes	NR NR NR NR NR	NR NR NR NR NR	NR NR NR NR NR	NR NR NR NR NR	NA NA NA NA Complete, transverse
**Bodur (2012) (see Supplementary Material)**	Left superior and inferior pubic ramus Right superior and inferior pubic ramus	Yes Yes	Yes Yes	NR NR	NR NR	NR NR	NR NR	Complete, transverse Complete, transverse
**McMahon (2009)^35^**	Right proximal humerus	Yes	Yes	Incomplete	No	Cortical thickening	No	Partial, lateral cortex origin
**Yavropoulou (2011) (see Supplementary Material)**	Humeral shaft	NR	No	NR	NR	NR	NR	NA
**Reichmister (2012) (see Supplementary Material)**	Case 1: left second metatarsal, rib Case 3: left metatarsal (site not specified) Case 5: 2x metatarsals and sacrum (specific site/side not specified)	Yes NR NR	No No No	NR NR NR	NR NR NR	NR NR NR	NR NR NR	NA NA NA
**Lopez (2012) (see Supplementary Material)**	L5 pedicle	Yes	No	NR	NR	NR	NR	NA
**Sietsema (2012) (see Supplementary Material)**	Humeral diaphysis (side not specified)	NR	No	NR	NR	NR	NR	NA
**Nacir (2013) (see Supplementary Material)**	Right inferior pubic ramus Right medial acetabular wall	Yes Yes	No No	NR NR	NR NR	NR NR	No No	NA NA
**Askin (2015)^20^**	Left iliac bone Bilateral sacral wings	Yes Yes	No (MRI) No (MRI)	NR NR	NR NR	Bone marrow oedema Bone marrow oedema	No No	NA NA
**Varghese (2018) (see Supplementary Material)**	Left inferior and superior pubic rami Right sacral ala	Yes Yes	No No	NR NR	NR NR	NR NR	NR NR	NA NA
**Meleger (2020) (see Supplementary Material)**	L5 pedicle	Yes	Yes	Complete	NR	NR	No	Complete, transverse
**Sabsuantang (2021)^41^**	Proximal left ulna	No	Yes	Incomplete	NR	Cortical thickening, sclerosis	NR	Complete, transverse
**Suthar (2021) (see Supplementary Material)**	Left tibial plateau	No	Yes	NR	No	NR	NR	Partial, transverse
**Min (2022) (see Supplementary Material)**	Right 5th metatarsal	Yes	Yes	Incomplete	No	NR	No	Partial, transverse, lateral cortex origin
**Balikci (2022)^21^**	Bilateral femoral neck	Yes	Yes	Incomplete	No	NR	NR	Partial, transverse
**Breglia (2010) (see Supplementary Material)**	Left proximal tibial diaphysis	No	Yes	Incomplete	No	Severe osteopenia	No	Partial, presence of spike
**El Rachkidi (2011) (see Supplementary Material)**	L5 pedicle	Yes	Yes	Complete	NR	NR	NR	Complete, transverse
**Stathopoulos (2011) (see Supplementary Material)**	Right ulna	No	No (post-operative)	NR	NR	NR	NR	NA
**Waterman (2011)^46^**	Case 1: left 2nd metatarsal Case 2: left 5th metatarsal Case 3: left 5th metatarsal Case 4: bilateral 4th metatarsal Case 5: right 5th metatarsal Case 6: right 4th & 5th metatarsal	No No No No No No	No Yes No (MRI) No No No	NR NR NR NR NR NR	NR NR NR NR NR NR	NR NR NR NR NR NR	NR NR NR NR NR NR	NA Partial, transverse, lateral cortex origin NA NA NA NA
**Bjorgul (2011)^24^**	Left ulna	No	Yes	NR	No	Sclerosis	NR	Partial, transverse
**Imbuldeniya (2012)^32^**	Bilateral proximal tibia Left distal femur	Yes Yes	Yes No (healing)	NR NR	No No	Cortical thickening Cortical thickening	No No	Partial, transverse NA
**Pradhan (2012) (see Supplementary Material)**	Right 5th metatarsal shaft	Yes	Yes	Incomplete	NR	Cortical thickening	NR	Partial, transverse, lateral cortex origin
**Vun (2014) (see Supplementary Material)**	Right clavicle	No	Yes	NR	No	Cortical thickening	Yes	Complete, transverse fracture, presence of spike
**Haque (2016)^30^**	Left scapula (acromion)	No	Yes	NR	NR	NR	NR	Complete, transverse
**Khan (2016) (see Supplementary Material)**	Case 1: right intracapsular neck of femur Case 2: left intracapsular neck of femur	No Yes	Yes Yes	Complete Incomplete	No No	NR NR	NR NR	Complete, transverse Partial, transverse
**Erdem (2016)^29^**	Right proximal ulna	No	Yes	Complete	No	Sclerosis	No	Complete, transverse
**Ang (2013)^19^**	Bilateral ulna	NR	Yes	NR	No	Sclerosis	NR	Complete, transverse
**Moon (2013)^36^**	Case 1: left proximal ulna Case 2: left radius	Yes Yes	Yes Yes	NR NR	NR NR	Sclerosis, cortical thickening Sclerosis, cortical thickening	NR NR	Partial, transverse Partial, transverse
**Patel (2013) (see Supplementary Material)**	Left superior and inferior pubic rami	Yes	Yes	Complete	No	NR	Yes	Complete, transverse
**Bissonnette (2013)^23^**	Left tibial diaphysis	Yes	Yes	Complete	No	Cortical thickening	Yes	Complete, transverse
**Dandinoglu (2014) (see Supplementary Material)**	Right humerus	No	Yes	Complete	No	No	NR	Complete, transverse
**Chiang (2014)^28^**	Right ulna	Yes	Yes	Complete	No	Sclerosis	NR	Complete, transverse
**Osada (2015) (see Supplementary Material)**	Left ulna	Yes	Yes	Complete	NR	Cortical thickening, sclerosis	NR	Complete, transverse
**Kim (2015) (see Supplementary Material)**	L4 pedicle	Yes	No (SPECT–CT)	NR	NR	NR	NR	NA
**Karabay (2015) (see Supplementary Material)**	L1-L4 pedicle	Yes	No (CT)	Complete	NR	NR	NR	NA
**Lim (2015)^33^**	Bilateral tibial stress reaction	Yes	Yes	Incomplete	No	MRI T2 hyperintensity	NR	Partial (only visible on MRI)
**Ayanaoglu (2015)^48^**	Left pubic rami Bilateral sacral ala	NR NR	No (MRI) No (MRI)	NR NR	NR NR	Bone marrow oedema Bone marrow oedema	NR NR	NA NA
**Murray (2016)^38^**	Bilateral distal fibula	Yes	Yes	NR	NR	NR	NR	Complete, transverse
**Martin Arias (2017) (see Supplementary Material)**	Sternum (Upper manubrium, lower manubrium, and body)	No	No (MRI)	NR	No	NR	No	NA
**Kwak (2017) (see Supplementary Material)**	Case 1: right pubic ramus, right sacral wing, left medial acetabular wall, left femoral head Case 2: left medial acetabular wall	Yes Yes	Yes Yes	NR NR	NR NR	NR NR	NR NR	NA NA
**Shimada (2017)^42^**	Case 1: right ulna Case 2: left ulna	Yes Yes	Yes Yes	NR NR	No No	Cortical thickening, sclerosis Sclerosis	NR NR	Complete, transverse Complete, transverse
**Malabu (2019)^34^**	Right proximal tibial stress	Yes	Yes	NR	No	No	No	Partial, transverse
**Alwahhabi (2017)^17^**	3 patients had tibial fracture 1 patient had fibula fracture 1 patient had pelvic fracture	NR NR NR	Yes Yes Yes	NR NR NR	NR Yes NR	NR NR NR	NR NR NR	Transverse Transverse NA
**Yam (2017)^47^**	Left ulna Right ulna	No No	Yes Yes	NR Incomplete	No No	Cortical thickening Cortical thickening	NR NR	Partial, transverse, lateral cortex origin Partial, transverse, lateral cortex origin
**Oh (2018) (see Supplementary Material)**	Left ulna	No	Yes	Complete	No	Cortical thickening	NR	Complete, transverse, presence of spike
**Surur (2018)^43^**	L5 pedicle	Yes	Yes	NR	NR	NR	NR	Complete, transverse
**Tan (2019)^44^**	Bilateral tibia	No	Yes	Incomplete	No	NR	NR	Partial, transverse
**Asano (2020)^18^**	Left ulna Right ulna	No No	Yes Yes	Complete Incomplete	No No	Cortical thickening, sclerosis Cortical thickening, sclerosis	No No	Complete, transverse Partial, transverse
**Abe (2020)^16^**	Left ulna	Yes	Yes	Complete	No	Cortical thickening, sclerosis	NR	Complete, transverse
**Cha (2021)^27^**	Case 1: left ulna Case 2: right ulna Case 3: left ulna Case 4: left ulna	Yes Yes Yes Yes	Yes No Yes Yes	Complete Complete Complete Complete	No No No No	Cortical thickening, sclerosis Cortical thickening, sclerosis Cortical thickening, sclerosis Cortical thickening, sclerosis	No No No No	Complete, transverse NA Complete, transverse Complete, transverse
**Koiwai (2021) (see Supplementary Material)**	Left distal femur Left tibia	NR NR	Yes Yes	NR NR	NR NR	Sclerosis Sclerosis	No No	Partial, transverse Partial, transverse
**Binkley (2021)^22^**	Right ulna	Yes	Yes	Incomplete	No	NR	Yes	Partial, transverse
**Cha (2021)^26^**	Radius (1 x patient) Ulna (17 x patients) Radio-ulna (2 x patients) Bilateral forearm (4 x patients)	NR NR NR NR	Not for specific cases	NR NR NR NR	NR NR NR NR	NR NR NR NR	NR NR NR NR	NA NA NA NA
**Hatano (2021) (see Supplementary Material)**	Left ulna	Yes	Yes	NR	No	NR	NR	Transverse
**Murai (2021)^37^**	Right ulna	NR	Yes	Incomplete	No	Cortical thickening, sclerosis	Yes	Partial, transverse
**Ohta (2022)^39^**	Bilateral ulna	No	Yes	NR	No	Cortical thickening, sclerosis	NR	Complete, transverse
**Kim (2020) (see Supplementary Material)**	Right femoral neck	Yes	Yes	Incomplete	No	NR	No	Partial, transverse
**Okamoto (2022)^40^**	Left ulna	No	Yes	Complete	No	Cortical thickening	NR	Complete, transverse
**Heo (2022)^31^**	Case 1: right ulna Case 2: right ulna Case 3: left ulna Case 4: bilateral ulna Case 5: left ulna Case 6: left ulna Case 7: left ulna Case 8: bilateral ulna Case 9: right ulna Case 10: left ulna	No No No No No No No Yes No No	No Yes No No No No No No No No	Complete Complete Complete Complete Complete Complete Complete Complete/incomplete Complete Complete	No No No No No No No No No No	Cortical thickening Cortical thickening NR Cortical thickening NR Cortical thickening NR Cortical thickening NR Cortical thickening	Yes Yes Yes Yes Yes Yes Yes Yes Yes Yes	NA Complete, transverse, presence of spike NA NA NA NA NA NA NA NA
**Ilyas (2022) (see Supplementary Material)**	Left scapular spine	No	Yes	NR	NR	Cortical thickening, vertical fracture pattern	No	Partial, transverse
**Nakajima (2022) (see Supplementary Material)**	Left radial diaphysis	Yes	Yes	Incomplete	NR	Cortical thickening, sclerosis	NR	Partial, transverse
**Odvina (2005)^9^**	Case 1: sacrum Case 2: vertebra, rib Case 5: sacrum, ischium Case 6: pubic rami Case 9: metatarsal	NR NR NR NR NR	No No No No No	NR NR NR NR NR	NR NR NR NR NR	NR NR NR NR NR	NR NR NR NR NR	NA NA NA NA NA

^*^evaluated by reviewers L.C. and A.R.

Abbreviations: NR, not reported; NA, not applicable; MRI, magnetic resonance imaging; CT, computerized tomography; SPECT CT, single-photon emission computed tomography

Mobility status was reported in 53 individuals prior to their atypical fracture: 41 were independent, nine required a walking aid (frame/cane/crutch), and three used a wheelchair. Of the individuals that required a walking aid (*n* = 9), all sustained upper arm fractures (eight ulnar fractures^19^^,^^24^^,^^28^^,^^29^^,^^37^^,^^39^^,^^41^^,^^47^ and one radial fracture^36^). While not utilizing a walking aid, a right-sided ulnar fracture was reported in a female who used a wheelchair, who used her right arm to support herself when transferring from her wheelchair.^45^ Interestingly, one case reported a left ulnar fracture in a patient who lent on her left arm for support during kitchen work due to her severe spinal kyphosis.^40^

Fracture characteristics are presented in Table 3 (see Supplementary Material). Of the 151 atypical fractures included in this review, radiographs were provided in 74 (49%) and reviewed by authors L.C. and A.R. Seventy fractures were transverse in nature (95%). One was unable to be visualized clearly in the attached radiograph,^17^ and the two tibial stress fractures described by Lim and colleagues were only subtly visible on the MRI, but could not be seen on the included radiographs.^33^ The right humeral fracture described by McMahon and colleagues was a longitudinal stress fracture running parallel to the long axis of the humerus.^35^ Partial and complete fractures were identified in 31 (42%) and 40 (54%) cases, respectively.

There was significant variability in the detail of fracture characteristics described by authors. Non-comminuted fractures were reported in 60/61 cases (98%). Cortical thickening was noted in the bone surrounding 36/52 fractures (69%). The presence of beaking of the lateral cortex was reported in 18/27 fractures (67%).

Bilateral/contralateral fractures were reported in 19% of cases, occurring at a variety of sites including the ulna,^18^^,^^19^^,^^26^^,^^31^^,^^39^^,^^47^ tibia,^32^^,^^33^^,^^44^ fibula,^38^ sacrum,^20^^,^^48^ metatarsal,^46^ and the femoral neck.^21^

Due to the variety of fractures in this review, the management and healing outcomes for the most frequent fracture, ulna, were reviewed (Table 4). Ulnar fractures were managed either conservatively (*n* = 6) or surgically (*n* = 32). Management was not reported in 21 cases. Of the conservatively managed ulnar fractures, four cases were immobilized with long arm casting (67%). The outcome of these cases is unclear; three patients were lost to follow-up and one case was not reported. Two of the conservatively managed cases included the addition of teriparatide therapy. One teriparatide-treated case involved an incomplete ulnar fracture that achieved union after 4 mo of treatment.^22^ The second case did not achieve union despite teriparatide treatment.^41^ Only two of the conservatively managed ulnar fractures achieved union,^18^^,^^22^ with the median duration to union being 8 mo (IQR 6-10).

**Table 4:** Treatment and healing outcomes for atypical ulnar fractures.

	No. of cases (%)
**Management approach**	
**Conservative management**	6 out of 38 cases (15.8%)
**Surgical intervention**	32 out of 38 cases (84.2%)
**Teriparatide treatment**	5 out of 38 cases (13.2%)
**Cessation of anti-resorptive therapy**	36 out of 40 cases (90%)
**Healing outcomes**	
**Delayed healing (>6 mo)**	16 out of 28 cases (57.1%)
Duration of healing^*^ (mo, median (IQR))	7.5 (5-12)

^*^Data available for 28 cases.

Abbreviations: IQR; interquartile range.

Thirty-two ulnar fractures were managed surgically; nine cases required re-operation (of those, three patients refused redo surgery). A variety of surgical methods were utilized, including plating, bone substitute, intramedullary nail, autologous cancellous bone grafting (ACBG), and open reduction and internal fixation (ORIF). Four cases refused surgical intervention. Three cases utilized teriparatide in addition to surgical intervention (ORIF, bone graft, and low-intensity pulsed ultrasound (LIPUS)^16^^,^^42^); all achieved fracture union. Union was achieved in 25 cases (six cases required re-operation to achieve fracture union).^27^^,^^42^ Non-union was reported in five cases (three of these cases refused redo surgery). Outcome was not reported in two cases. Of the ulnar fractures that achieved union, the median time to fracture union was 8 mo (IQR 5.5-12).

Laboratory investigations and BMD results as assessed by dual-energy X-ray absorptiometry (DXA) were reviewed (Table 5). The median calcium, 25OHD, and creatinine values were within normal limits. The overall median *L*_1_-*L*_4_ BMD T-score was −2.4, similar to the median *L*_1_–*L*_4_ BMD T-score of those who sustained an ulnar fracture.

**Table 5:** Summary of investigations; biochemistry and BMD results as assessed by DXA.

	**All fractures**	**Ulna fractures**	**Reference range**
**Calcium (mmol/L, median (IQR))**	2.33 (2.28-2.41) (*n* = 23)	2.32 (2.23-2.38) (*n* = 7)	2.10-2.60 mmol/L
**25-hydroxyvitamin D (nmol/L, median (IQR))**	88.4 (54.5-115.6) (*n* = 26)	78.5 (38.8-114.0) (*n* = 6)	25-150 nmol/L
**Creatinine (mmol/L, median (IQR))**	70.7 (54.8-84.0) (*n* = 15)	52.2 (49.5-57.5) (*n* = 4)	49-90 mmol/L
**L_1_-L_4_ BMD T-score (median (IQR))**	−2.4 (−3.1-−1.5) (*n* = 58)	−2.4 (−3.1-−1.5) (*n* = 25)	NA
**FN BMD T-score (median (IQR))**	−2.0 (−2.7--−1.2) (*n* = 36)	−2.8 (−3.1-−2.1) (*n* = 7)	NA
**TH BMD T-score (median (IQR))**	−1.3 (−1.8-−0.8) (*n* = 11)	−1.1 (−2.05-−0.2) (*n* = 4)	NA

Abbreviations: FN, femoral neck; TH, total hip; NA, not applicable.

Bone turnover markers were reported in a minority of included articles (Table 6, see Supplementary Material). Thirteen articles, describing 17 individuals, reported a value for a marker of bone resorption, either urinary collagen cross-linked N-telopeptide (NTx) or C-terminal telopeptide of type 1 collagen (CTx). CTx and NTx values were in the lower third or below the reference range in eight reports (47%).

**Table 6:** Bone turnover markers.

**Author (year)**	**CTx (reference range)**	**NTx (reference range)**	**P1NP (reference range)**
**Malabu (2019)^34^**	430 ng/L (<800)	49 μg/L (15-90)	NR
**Kwak (2017) (see Supplementary Material)**	Case 1: 0.495 ng/mL (0.01-1.00) Case 2: 0.140 ng/mL (0.01-1.00)	NR	NR
**Kim (2012) (see Supplementary Material)**	0.33 ng/mL (0.01-1.00)	NR	NR
**Kim (2020) (see Supplementary Material)**	NR	8 nmol BCE/nmol Cr (11-91)	NR
**Murray (2016)^38^**	0.132 ng/mL (0.05-0.6)	NR	NR
**Suthar (2021) (see Supplementary Material)**	0.195 pg/L (nil reference range)	NR	32.5 μg/L (nil reference range)
**Breglia (2010) (see Supplementary Material)**	19 nmol BCE/nmol Cr (4-64)	NR	NR
**Bissonnette (2013)^23^**	0.271 μg/L (0.104–1.008)	NR	NR
**Lim (2015)^33^**	NR	12 nmol/mmol (21-66)	NR
**Oh (2018) (see Supplementary Material)**	0.593 ng/mL (<1.00)	NR	NR
**Binkley (2021)^22^**	NR	28.2 nmol BCE (5.4-24.2)	NR
**Okamoto (2022)^40^**	NR	45.6 ng/mL (nil reference range)	NR
**Abe (2020)^16^**	NR	NR	15.5 ng/mL (26.4–98.2)
**Odvina (2005)^9^**	NR	Case 1: 53.3 nmol BCE/mmol Cr (5-65) Case 2: 8.8 nmol BCE/mmol Cr (5-65) Case 5: 24 nmol BCE/mmol Cr (5-65) Case 6: 31 nmol BCE/mmol Cr (5-65) Case 9: 28 nmol BCE/mmol Cr (5-65)	NR

Abbreviations: NR, not reported; BCE, bone collagen equivalents; CTx, C-terminal telopeptide of type 1 collagen; NTx, urinary cross-linked N-telopeptides of type 1 collagen; P1NP, procollagen type 1 N-propeptide.

## Discussion

Anti-resorptive therapy for osteoporosis in men and women is common and effective at preventing fragility fractures; however, prolonged use has been associated with an increased risk of AFFs,^12^^,^^49^ particularly in patients of Asian ethnicity. Although a rare event, an AFF is highly significant and devastating for an affected patient. The first reported association between anti-resorptive therapy and atraumatic atypical fractures was described in 2005 by Odvina and colleagues.^9^ The femur was the most common site; however, even in this seminal report, seven atypical fractures occurred at non-femoral sites. This systematic review supports the hypothesis that anti-resorptive therapy-related atypical fractures may also occur at non-classical sites and identified 151 atypical fractures in 114 published cases. The most frequent fracture site was the ulna, followed by the tibia. All patients were taking anti-resorptive treatment prior to/at the time of fracture, most frequently alendronate monotherapy (44%). Most commonly, fractures were transverse in nature (95%), following minimal/no trauma (96%), or non-comminuted (98%) with cortical thickening of the surrounding bone (69%). Anti-resorptive treatment was ceased following atypical fracture in most cases (89%). Like AFFs, repetitive force, prodromal pain, and delayed healing appear to be common in this cohort of atypical fractures at other skeletal sites. Given the association with anti-resorptive therapy and AFFs, it is unsurprising that atypical fractures may occur at non-classical sites with long-term anti-resorptive therapy.

Bisphosphonates have a high affinity for hydroxyapatite, allowing avid attachment to bony surfaces and internalization by osteoclasts. The viability of osteoclasts is reduced, leading to reduced bone resorption. While bisphosphonate use increases the strength of bone, it also increases the stiffness of bone via altering collagen maturity and cross-linking.^50^ Bisphosphonate treatment has been demonstrated to increase non-enzymatic pentosidine cross-links, leading to increased advanced glycation end products in bone, deteriorating the mechanical properties of bone and fracture resistance.^51^^,^^52^ In addition, bisphosphonate exposed bone is more brittle and has been shown to have reduced fatigue life (fewer cycles of stress before failure).^53^ Atypical fractures have also been reported in patients on denosumab, and we identified one case of bilateral atypical tibial stress reaction following 4 yr of denosumab monotherapy.^33^^,^^54^

Ulnar fractures were the predominant location highlighted in this review. The relationship between bone geometry and the site of fracture has been discussed in reports of AFFs. A more varus femorotibial angle and lateral femoral bowing place maximal tensile loading on the lateral femoral cortex, predisposing to AFFs.^12^ Repetitive force also appears to be important in the pathogenesis of AFFs, possibly due to the accumulation of microdamage. Bone remodeling suppression by anti-resorptive treatment further impedes healing of skeletal sites with microdamage. This review identified nine individuals that required a walking frame/cane/crutch for mobilization (thereby weight-bearing through their upper limbs). All sustained upper arm fractures, predominantly at the ulna. In addition, two further ulnar fractures were identified in elderly women who supported their weight through their upper limbs.^40^^,^^45^ For these patients, similar to the hypothesized pathogenesis of AFF, accumulation of microdamage following repetitive force, may have contributed to the development of the atypical fracture. However, we did identify 12 ulnar fractures in individuals independent in ambulation.^31^ Another mechanism, perhaps a genetic or ethnic predisposition, may contribute to the generation of these atypical fractures.

AFFs have been recognized and reported in bisphosphonate-naïve individuals, found to have an underlying monogenetic bone disorder, such as “osteogenesis imperfecta, hypophosphatasia, osteopetrosis, pycnodysostosis, X-linked hypophosphataemia, X-linked osteoporosis, and osteoporosis pseudoglioma syndrome.”^50^ Our review identified one atypical fracture in the setting of an underlying monogenetic bone disorder (hypophosphatasia). An atypical tibial fracture unmasked underlying hypophosphatasia following 4.5 yr of intermittent alendronate exposure.^34^ Indeed, a recent systematic review identified multiple gene variants in AFF populations, proposing that mild, unrecognized forms of monogenetic bone diseases may underlie the etiology of AFFs.^50^

The risk of developing an AFF is associated with Asian race.^55–57^ Lo and colleagues reported in 2016 an 8-fold higher rate of AFF among Asian women compared with White women. Although Asian women received slightly longer bisphosphonate treatment (median 3.8 vs 2.7 yr) compared with White women, after adjusting for bisphosphonate duration, the relative hazard for AFF in Asian women remained elevated at 6.6 (95% confidence interval 3.7-11.5). The unique femoral geometry of Asian women has been attributed to this difference. A shorter hip axis length and larger varus femorotibial angle and bowing contribute to increased stress on the lateral femur.^56^ Whether similar skeletal differences are apparent at other sites to the femur remains unclear at this stage. Fifty-nine ulnar fractures were included in this review. Of these, only five articles (describing six fractures) commented on ethnicity. Five ulnar fractures occurred in individuals of Asian ethnicity (Thai, Chinese, and Japanese). At this stage, it is not clear whether Asian ethnicity is also important in the pathogenesis of atypical fractures at non-classical sites, as it is in AFFs. Further research is required to ascertain whether ethnic-specific factors, such as unique geometry, differences in genetic predisposition, treatment patterns, drug adherence, and age of treatment, initiation contribute to atypical fracture risk at non-classical sites.^56^ Due to the elevated risk of AFF in Asian individuals, future case descriptions of atypical fractures at non-classical sites should strongly consider including ethnicity in their reporting.

This review highlights fracture characteristics integral to diagnosing an atypical fracture. Four of the five major features of an AFF utilizing the ASBMR case definition involve radiographic features of the fracture (Table 1). In this review, the most common atypical fracture characteristics were the transverse pattern (95%), absence of comminution (98%), and cortical beaking (67%) (Table 3, see Supplementary Material). Indeed, this is concordant with recently published reviews of atypical fractures.^13^^,^^14^ Conversely, only 5% of included radiographs demonstrated the presence of a cortical spike. This may be attributable to the fact that many of the fractures included in this review were not in weight-bearing bones.^14^ Prodromal pain, a minor feature of the AFF case definition, was common (62%). Fractures that bear a strong resemblance to the ASBMR AFF case definition should alert clinicians to the possibility of atypical fractures related to long-term anti-resorptive treatment.

Given the large cohort of heterogenous fractures identified in this review, examining treatment outcomes was challenging. Instead, the most common atypical fracture site was the focus of this review. Of the conservatively managed ulnar fractures, outcomes were only available in 3 of 7 cases (two achieved union, one did not achieve union). Predominantly, ulnar fractures were managed surgically, and subsequently achieved union (25 of 32 fractures). The ideal surgical approach remains unclear. Cha et al., reported outcomes of revision surgeries to treat non-union of atypical ulnar fractures that had originally been treated using ORIF.^27^ All four patients achieved union following revision surgery involving resection of non-union area, iliac bone graft, and re-fixation. Resection of the non-union area has been speculated to improve healing due to the low bone turnover state and suppressed bone remodeling following long-term anti-resorptive administration. Further studies are required to determine whether teriparatide and/or LIPUS are effective in the management of atypical fractures.

This study represents the first systematic review of all published cases of anti-resorptive-related atypical fractures that do not strictly fulfill the ASBMR case definition. One limitation of this study is the selection of articles written only in English and including articles without associated radiographic imaging of some/all the fractures described. Further, complete capture of all outcomes and fracture characteristics was difficult as articles were mainly case reports and heterogenous in their description. Second, although doubted by the authors of the primary literature, it is possible that the fractures in patients with a concurrent malignancy may have been pathologic in nature. Third, an assessment of the risk of bias was not conducted, as the included articles were predominantly case series and case reports. Furthermore, atypical fracture cases in anti-resorptive naïve individuals were excluded from this systematic review. We acknowledge the inherent selection bias in the publication of such reports; however, these case series and reports highlight a rising concern within the medical community that atypical fractures may occur at sites other than the femur. Larger cohort studies should be undertaken to assess the incidence of atypical fractures at non-classical sites, in particular at ulna sites, and to elucidate risk factors and their relationship with anti-resorptive treatment.

Atypical fractures are rare and should not deter clinicians from starting appropriate anti-resorptive therapy for patients with osteoporosis or at high risk of fragility fracture. Fractures that occur while a patient is receiving anti-resorptive therapy should be evaluated for atypical features. Fracture characteristics, such as being sustained following no/minimal trauma, prodromal pain, a history of anti-resorptive treatment (typically >3 yr), transverse and absent comminution, regardless of site, should raise suspicion. Similar to Shane and colleagues, we recommend, if confirmed to be an atypical fracture in the setting of anti-resorptive therapy, to consider anti-resorptive therapy cessation, limit weight-bearing, consider surgical management, and manage their underlying osteoporosis.^11^^,^^12^ The role of anabolic therapies, teriparatide and romosozumab, remains unclear at this stage, and further studies are required.

## Conclusion

At present, the ASBMR case definition of AFF is solely limited to fractures observed in the femur, originating at the lateral cortex. The reports of possible atypical fractures at sites excluded from the ASBMR AFF case definition in patients receiving long-term anti-resorptive therapy are important to recognize and highlight. Clinicians should be aware that atypical fractures can occur at sites other than the femur. Considering the findings from this study, as well as the recent systematic review on periprosthetic AFFs, it may be timely to review and update the current ASBMR AFF case definition.

## Supplementary Material

Search_Strategy_zjae159

Supplemenetary_Reference_List_zjae159

## Data Availability

The data pertaining to our search strategy are available in the article and in its online supplementary material. Additional data underlying this article will be shared on reasonable request to L.C., collins.lucy92@gmail.com
