# Supplementary material for: Atypical fractures at non-classical sites associated with anti-resorptive therapy: a systematic review
Source: J Bone Miner Res. 2024 Sep 30;39(12):1722–34. doi: 10.1093/jbmr/zjae159 (PMC11638334; doi:10.1093/jbmr/zjae159)
Supplement: Search_Strategy_zjae159 [file search_strategy_zjae159.pdf]

**Medline (Ovid) – FINAL Search Strategy – As at Friday, 21 October 2022**

-----------------------------

Search for: 11 and 20

Database: Ovid MEDLINE(R) and Epub Ahead of Print, In-Process, In-Data-Review & Other Non-Indexed Citations and Daily
<1946 to October 19, 2022>
Search Strategy:
--------------------------------------------------------------------------------
1 bone density conservation agents/ (15741)
2 (antiresorptive* or anti-resorptive* or anti resorptive* or bone density conserv* or bone resorption).mp. (56853)
3 exp Diphosphonates/ (27837)
4 Denosumab/ (2242)
5 Alendronate/ (3989)
6 Ibandronic Acid/ (764)
7 Pamidronate/ (2165)
8 Zoledronic Acid/ (3923)
9 Risedronic Acid/ (1246)
10 (diphosphonate* or bisphosphonate* or denosumab* or prolia or xgeva or alendronate* or Ibandronic acid* or
pamidronate* or zoledronic acid* or zoledronate* or zometa or aclasta or risedronic acid* or actonel or risedronate* or atelvia or ibanaronate* or bondronat or boniva or bonviva or romosozumab*).mp. (36843)
11 1 or 2 or 3 or 4 or 5 or 6 or 7 or 8 or 9 or 10 (82194)
12 exp Fractures, Bone/ (203432)
13 fractur*.ti,ab. (293777)
14 12 or 13 (340301)
15 (atypical or insufficiency or stress).ti,ab. (1136591)
16 14 and 15 (19352)
17 Fractures, Stress/ (3747)
18 (atypical fracture* or insufficiency fracture* or stress fracture*).ti,ab. (5975)
19 (atypical or insufficiency or stress).ti,ab. (1136591)
20 16 or 17 or 18 or 19 (1137524)
21 11 and 20 (3863)

***************************

---------------------------------------------------------------------------------------------------------------------------------------------
--

**Embase – FINAL Search Strategy - As at Friday, 21 October 2022**

-----------------------------

Search for: 12 and 22

Database: Embase <1974 to 2022 October 19> Search Strategy:

--------------------------------------------------------------------------------
1 bone density conservation agent/ (4006)
2 (antiresorptive* or anti-resorptive* or anti resorptive* or bone density conserv* or bone resorption).mp. (45561)
3 exp bisphosphonic acid derivative/ (74434)
4 denosumab/ (11688)
5 alendronic acid/ (17889)
6 ibandronic acid/ (5771)
7 pamidronic acid/ (11145)
8 zoledronic acid/ (18973)
9 risedronic acid/ (8453)
10 romosozumab/ (924)
11 (diphosphonate* or bisphosphonate* or denosumab* or prolia or xgeva or alendronate* or Ibandronic acid* or
pamidronate* or zoledronic acid* or zoledronate* or zometa or aclasta or risedronic acid* or actonel or risedronate* or atelvia or ibanaronate* or bondronat or boniva or bonviva or romosozumab*).mp. (60937)
12 1 or 2 or 3 or 4 or 5 or 6 or 7 or 8 or 9 or 10 or 11 (118640)
13 exp fracture/ (334606)
14 fractur*.ti,ab. (347423)
15 13 or 14 (436358)
16 (atypical or insufficiency or stress).ti,ab. (1435777)
17 15 and 16 (25270)
18 insufficiency fracture/ (153)
19 stress fracture/ (7212)
20 (atypical fracture* or insufficiency fracture* or stress fracture*).ti,ab. (7477)
21 (atypical or insufficiency or stress).ti,ab. (1435777)
22 17 or 18 or 19 or 20 or 21 (1438175)
23 12 and 22 (6045)

***************************

---------------------------------------------------------------------------------------------------------------------------------------------
---------------------------------------------------------------------------------------------------------------------------------------------
-

**Cochrane – FINAL Search Strategy - As at Friday, 21 October 2022**

**Cochrane - FINAL Search Strategy - As at Friday, 21 October 2022**

Last saved on: 21/10/2022 13:28:03

✔ Search saved.

View fewer lines | Print search history

| # | Search | | Results |
|---|---|---|---|
| #1 | MeSH descriptor: [Bone Density Conservation Agents] this term only | MeSH | 1699 |
| #2 | (antiresorptive* or anti-resorptive* or "anti resorptive" or "anti resorptives" or "bone density conservation" or "bone resorption"):ti (Word variations have been searched) | S Limits | 382 |
| #3 | (antiresorptive* or anti-resorptive* or "anti resorptive" or "anti resorptives" or "bone density conservation" or "bone resorption"):ab (Word variations have been searched) | S Limits | 3177 |
| #4 | MeSH descriptor: [Diphosphonates] explode all trees | MeSH | 2693 |
| #5 | MeSH descriptor: [Denosumab] this term only | MeSH | 404 |
| #6 | MeSH descriptor: [Alendronate] this term only | MeSH | 778 |
| #7 | MeSH descriptor: [Ibandronic Acid] this term only | MeSH | 206 |
| #8 | MeSH descriptor: [Pamidronate] this term only | MeSH | 243 |
| #9 | MeSH descriptor: [Zoledronic Acid] this term only | MeSH | 671 |
| #10 | MeSH descriptor: [Risedronic Acid] this term only | MeSH | 263 |
| #11 | (diphosphonate* or bisphosphonate* or denosumab* or prolia or xgeva or alendronate* or "Ibandronic acid" or "Ibandronic acids" or pamidronate* or "zoledronic acid" or "zoledronic acids" or zoledronate* or zometa or aclasta or "risedronic acid" or "risedronic acids" or actonel or risedronate* or atelvia or ibanaronate* or bondronat or boniva or bonviva or romosozumab*):ti,ab,kw (Word variations have been searched) (Word variations have been searched) | S Limits | 6482 |
| #12 | #1 or #2 or #3 or #4 or #5 or #6 or #7 or #8 or #9 or #10 or #11 | Limits | 9338 |
| #13 | MeSH descriptor: [Fractures, Bone] explode all trees | MeSH | 6916 |
| #14 | (fractur*):ti (Word variations have been searched) | S Limits | 12990 |
| #15 | (fractur*):ab (Word variations have been searched) | S Limits | 21151 |
| #16 | #13 or #14 or #15 | Limits | 24800 |
| #17 | (atypical or insufficiency or stress):ti (Word variations have been searched) | S Limits | 24298 |
| #18 | (atypical or insufficiency or stress):ab (Word variations have been searched) | S Limits | 81733 |
| #19 | #17 or #18 | Limits | 88359 |
| #20 | #16 and #19 | Limits | 1298 |
| #21 | MeSH descriptor: [Fractures, Stress] this term only | MeSH | 117 |
| #22 | ("atypical fracture" or "atypical fractures" or "insufficiency fracture" or "insufficiency fractures" or "stress fracture" or "stress fractures"):ti (Word variations have been searched) | S Limits | 80 |
| #23 | ("atypical fracture" or "atypical fractures" or "insufficiency fracture" or "insufficiency fractures" or "stress fracture" or "stress fractures"):ab (Word variations have been searched) | S Limits | 225 |
| #24 | (atypical or insufficiency or stress):ti (Word variations have been searched) | S Limits | 24298 |
| #25 | (atypical or insufficiency or stress):ab (Word variations have been searched) | S Limits | 81733 |
| #26 | #20 or #21 or #22 or #23 or #24 or #25 | Limits | 88395 |
| #27 | #12 and #26 | Limits | 371 |

-------------------------------------------------------------------------------------------------------------------------------------------------------

-------------------------------------------------------------------------------------------------------------------------------------------------------

--

## Web of Science – FINAL Search Strategy - As at Friday, 21 October 2022

0/28 Combine Sets Export Clear History

| | | | |
|---|---|---|---|
| 28 | #13 AND #27 | 3,105 | Add to query |
| 27 | #21 OR #22 OR #23 OR #24 OR #25 OR #26 | 2,066,158 | Add to query |
| 26 | AB=(atypical or insufficiency or stress) | 1,793,073 | Add to query |
| 25 | TI=(atypical or insufficiency or stress) | 651,019 | Add to query |
| 24 | AB=("atypical fracture*" or "insufficiency fracture*" or "stress fracture*") | 4,104 | Add to query |
| 23 | TI=("atypical fracture*" or "insufficiency fracture*" or "stress fracture*") | 2,998 | Add to query |
| 22 | TS=(Stress fracture) | 118,655 | Add to query |
| 21 | #17 AND #20 | 90,563 | Add to query |
| 20 | #18 OR #19 | 2,053,236 | Add to query |
| 19 | AB=(atypical or insufficiency or stress) | 1,793,073 | Add to query |
| 18 | TI=(atypical or insufficiency or stress) | 651,019 | Add to query |
| 17 | #14 OR #15 OR #16 | 523,998 | Add to query |
| 16 | AB=(fractur*) | 411,088 | Add to query |
| 15 | TI=(fractur*) | 228,324 | Add to query |
| 14 | TS=(Bone fractures) | 106,158 | Add to query |
| 13 | #12 OR #11 OR #10 OR #9 OR #8 OR #7 OR #6 OR #5 OR #4 OR #3 OR #2 OR #1 | 68,272 | Add to query |
| 12 | ALL=(diphosphonate* or bisphosphonate* or denosumab* or prolia or xgeva or alendronate* or Ibandronic acid* or pamidronate* or zoledronic acid* or zoledronate* or zometa or aclasta or risedronic acid* or actonel or risedronate* or atelvia or ibanaronate* or bondronat or boniva or bonviva or romosozumab*) | 45,130 | Add to query |
| 11 | TS=(Romosozumab) | 468 | Add to query |
| 10 | TS=(Risedronic Acid) | 66 | Add to query |
| 9 | TS=(Zoledronic Acid) | 9,653 | Add to query |

| | | | |
|---|---|---|---|
| 8 | TS=(Pamidronate) | 4,930 | Add to query |
| 7 | TS=(Ibandronic Acid) | 92 | Add to query |
| 6 | TS=(Alendronate) | 10,689 | Add to query |
| 5 | TS=(Denosumab) | 5,215 | Add to query |
| 4 | TS=(Diphosphonate*) | 5,188 | Add to query |
| 3 | AB=(antiresorptive* or anti-resorptive* or "anti resorptive*" or "bone density conserv*" or "bone resorption") | 24,930 | Add to query |
| 2 | TI=(antiresorptive* or anti-resorptive* or "anti resorptive*" or "bone density conserv*" or "bone resorption") | 6,719 | Add to query |
| 1 | TS=(bone density conservation agents) | 52 | Add to query |
