# Supplementary material for: Atypical fractures at non-classical sites associated with anti-resorptive therapy: a systematic review
Source: J Bone Miner Res. 2024 Sep 30;39(12):1722–34. doi: 10.1093/jbmr/zjae159 (PMC11638334; doi:10.1093/jbmr/zjae159)
Supplement: Supplemenetary_Reference_List_zjae159 [file supplemenetary_reference_list_zjae159.docx]

Supplemenetary Reference List

1. Bodur H, Konca S, Yilmaz O, Delialioglu OM, Gunel U. Multiple Fractures Associated With Alendronate Use. Turk J Phys Med. 2012;58(1):66-8.
2. Breglia MD, Carter JD. Atypical insufficiency fracture of the tibia associated with long-term bisphosphonate therapy. J Clin Rheumatol. 2010;16(2):76-8.
3. Dandinoglu T, Akarsu S, Karadeniz M, Tekin L, Aribal S, Kiralp MZ. Can long-term bisphosphonate use causes low-energy fractures? A case report. Osteoporos Int. 2014;25(2):773-6.
4. El Rachkidi R, Sari-Leret M-L, Wolff S. Atypical bilateral pedicle fracture in long-term bisphosphonate therapy. Spine. 2011;36(26):E1769-73.
5. Gharanizadeh K, Ravanbod H, Aminian A, Hatami S, Chaleshtori AS, Kazerani S. Acetabular and sacral insufficiency fractures in a patient with a long-term history of Alendronate consumption; a case report. BMC Musculoskelet Disord. 2023;24(1):211.
6. Hatano M, Kitajima I, Yamamoto S, Nakamura M, Isawa K, Hirota Y, et al. Case report: Osteomalacia due to bisphosphonate treatment in a patient on hemodialysis. BMC Nephrol. 2021;22(1):298.
7. Ilyas G, Senyuva G. A Scapular Spine Fracture Defined on the Basis of a Bisphosphonate: a Case Report and Review of the Literature. Acta Chir Orthop Traumatol Cech. 2022;89(4):312-4.
8. Karabay N, Ozer E, Ada E. Multi-level, bilateral pedicle fractures: case report. Turk Neurosurg. 2015;25(2):340-3.
9. Khan SK, Savaridas T, Hemers JS, Maarouf Z, Orgee JM, Orr MM. Atraumatic intracapsular neck of femur fractures after prolonged bisphosphonate treatment: a new atypical variant? Clin Cases Miner Bone Metab. 2016;13(1):38-41.
10. Kim DH, Lee EC, Kang SK. Insufficiency fracture of ipsilateral femur neck in patient treated with long term bisphosphonate treatment - a case report. J Bone Metab. 2012;19(2):159-62.
11. Kim H-S, Jung HY, Kim M-O, Joa K-L, Kim YJ, Kwon S-Y, et al. Successful conservative treatment: multiple atypical fractures in osteoporotic patients after bisphosphate medication: a unique case report. Medicine. 2015;94(5):e446.
12. Kim K-K, Park Y-W, Kim T-H, Seo K-D. Atypical femoral neck fracture after prolonged bisphosphonate therapy. J Pathol Transl Med. 2020;54(4):346-50.
13. Koiwai H, Kamimura M, Nakamura Y, Takahashi J, Taguchi A. Same-side insufficiency fractures of the tibia and femur after denosumab discontinuation: a case report. Mod Rheumatol Case Rep. 2021;5(1):178-81.
14. Kwak S-J, Cho Y-J, Jung G-Y, Lee J-H, Chun Y-S, Rhyu K-H. Acetabular Insufficiency Fracture Following Prolonged Alendronate Use and the Failure of Total Hip Arthroplasty in "Frozen" Bone: Two Cases Report. Hip Pelvis. 2017;29(4):286-90.
15. Lopez E, Meleger AL. Nontraumatic bilateral pedicle fractures in the setting of biphosphonate therapy: A case report. PM and R. 2012;4(10 SUPPL. 1):S317.
16. Martin Arias LH, Garcia Ortega P, Sainz Gil M, Navarro Garcia E, Treceno Lobato C, Delgado Armas V. Atypical Fracture of the Sternum After Long-Term Alendronate Plus Cholecalciferol Treatment: A Case Report. Drug Saf Case Rep. 2017;4(1):5.
17. Meleger AL, Lopez EE. Atraumatic bilateral lumbar pedicle fractures in a patient with back pain and history of long-term bisphosphonate therapy: A case report. Pain Physician. 2020;4(3):97-102.
18. Min BC, Chung CY, Park MS, Sung KH, Lee KM. A suspicious atypical fracture of 5th metatarsal bone: A case report. J Orthop Sci. 2022;27(1):281-3.
19. Nacir B, Genc H, Koca G, Gumusdat RP, Erdem HR. Insufficiency fractures of acetabular medial wall and inferior pubic ramus associated with the long term alendronate use. Turk J Phys Med Rehabil. 2013;59(SUPPL. 1):386.
20. Nakajima T, Ohno K, Yokota A, Yasuda E, Neo M. Atypical Fracture of Radial Diaphysis After Sauve-Kapandji Procedure: A Retrospective Radiographic Evaluation of Fracture Progression. Orthopedics. 2022(pcm, 7806107, okl):1-4.
21. Oh BH, Heo YM, Yi JW, Kim TG, Lee JS. Atypical Fracture of the Proximal Shaft of the Ulna Associated with Prolonged Bisphosphonate Therapy. Clin Orthop Surg. 2018;10(3):389-92.
22. Osada R, Zukawa M, Kimura T. Atypical ulnar fracture associated with long-term bisphosphonate use. J Orthop Sci. 2015;20(6):1132-5.
23. Patel V, Graves L, Lukert B. Pelvic fractures associated with long-term bisphosphonate therapy - case report. J Musculoskelet Neuronal Interact. 2013;13(2):251-4.
24. Pradhan P, Saxena V, Yadav A, Mehrotra V. Atypical metatarsal fracture in a patient on long term bisphosphonate therapy. Indian J Orthop. 2012;46(5):589-92.
25. Reichmister J, Jay P. Atypical fractures associated with long-term bisphosphonate use. Clin Geriatr. 2012;20(2):17-21.
26. Sietsema D, Jones C, Hoffmann M. Is bisphosphonate use associated with atypical humeral diaphyseal fractures? J Bone Min Res. 2012;27(SUPPL. 1).
27. Stathopoulos KD, Kosmidis C, Lyritis GP. Atypical fractures of the femur and ulna and complications of fracture healing in a 76-year-old woman with Sjogren's syndrome. J Musculoskeletal Neuronal Interact. 2011;11(2):208-11.
28. Suthar A, Yukata K, Suetomi Y, Yamazaki K, Sakai T, Fujii H. Bisphosphonate-related atypical insufficiency fracturof the tibial plateau: A case report. J Med Invest. 2021;68(1.2):186-8.
29. Varghese B, Falk C, Haroon NN. Atraumatic pelvic fractures associated with bisphosphonate therapy. Endocr Pract. 2018;24(Supplement 1):115-6.
30. Vun SH, Husami Y, Shareef S, Bramley D. Acute nontraumatic clavicle fracture associated with long-term bisphosphonate therapy. Case Rep Orthop. 2014;2014(101591806):986718.
31. Yavropoulou M, Giusti A, Ramautar S, Dijkstra S, Hamdy N, Papapoulos S. Atypical humeral fractures and bisphosphonate use. J Bone Min Res. 2011;26(SUPPL. 1).
